# CO_2_-induced pH reduction increases physiological toxicity of nano-TiO_2_ in the mussel *Mytilus coruscus*

**DOI:** 10.1038/srep40015

**Published:** 2017-01-05

**Authors:** Menghong Hu, Daohui Lin, Yueyong Shang, Yi Hu, Weiqun Lu, Xizhi Huang, Ke Ning, Yimin Chen, Youji Wang

**Affiliations:** 1 College of Fisheries and Life Science, Shanghai Ocean University, Shanghai 201306, China; 2 Zhejiang Provincial Key Laboratory of Organic Pollution Process and Control, Zhejiang University, Hangzhou 310058, China; 3 Department of Environmental Science, Zhejiang University, Hangzhou 310058, China

## Abstract

The increasing usage of nanoparticles has caused their considerable release into the aquatic environment. Meanwhile, anthropogenic CO_2_ emissions have caused a reduction of seawater pH. However, their combined effects on marine species have not been experimentally evaluated. This study estimated the physiological toxicity of nano-TiO_2_ in the mussel *Mytilus coruscus* under high pCO_2_ (2500–2600 μatm). We found that respiration rate (RR), food absorption efficiency (AE), clearance rate (CR), scope for growth (SFG) and O:N ratio were significantly reduced by nano-TiO_2_, whereas faecal organic weight rate and ammonia excretion rate (ER) were increased under nano-TiO_2_ conditions. High pCO_2_ exerted lower effects on CR, RR, ER and O:N ratio than nano-TiO_2_. Despite this, significant interactions of CO_2_-induced pH change and nano-TiO_2_ were found in RR, ER and O:N ratio. PCA showed close relationships among most test parameters, i.e., RR, CR, AE, SFG and O:N ratio. The normal physiological responses were strongly correlated to a positive SFG with normal pH and no/low nano-TiO_2_ conditions. Our results indicate that physiological functions of *M. coruscus* are more severely impaired by the combination of nano-TiO_2_ and high pCO_2_.

Increasing production of engineered nanoparticles (NPs) has raised concern about their potential biological and ecological effects^1^. Among nanomaterials, nanosized titanium dioxide (nano-TiO_2_) is considered as one of the most frequently used NPs and the most extensively investigated NP so far in ecotoxicology^2^. Nano-TiO_2_ has been released in the aquatic environments, such as the estuarine and coastal waters, causing a health risk to marine organisms^2^,^3^,^4^. Nano-TiO_2_ has been shown to induce detrimental biological effects on aquatic animals in different *in vitro* and *in vivo* systems^4^,^5^. To date, the potential impact of nano-TiO_2_ on aquatic ecosystems and marine invertebrates has attracted special attention^1^,^2^,^6^,^7^.

Marine bivalves are important model organisms to assess the effect of NPs on aquatic biota, and may play a significant role in NPs uptake, biotransformation and trophic transfer through food chains^8^,^9^,^10^. In general, nano-TiO_2_ has been shown to exert toxicity mainly via inducing ROS production and oxidative stress in cells^5^,^9^,^11^,^12^,^13^,^14^. Nano-TiO_2_ has showed toxic effects in bivalve hemocytes^15^,^16^,^17^,^18^, stimulating different immune responses (e.g., oxidative stress and the release of hydrolytic enzymes). TiO_2_ nanoparticles also displayed embryotoxicity to *Mytilus galloprovincialis*^19^ and induced DNA damage in *Lymnea luteola*^20^. Previous studies have demonstrated adverse/toxic effects of nano-TiO_2_ on immune system and digestive gland function in marine mussels^9^,^11^,^12^,^15^,^16^,^21^. In bivalves the gills represent the first site of contacting particles from the surrounding environment due to their role in the feeding process^22^. Katsumiti *et al*.^13^ found that nano-TiO_2_ showed adverse effects to mussel gill cells^13^. As gill cells play important roles in food transport and respiration for mussels^23^, there is a unique interest in expanding studies on nano-TiO_2_ toxicity in mussel’s feeding and physiology. However, information on the effects of nano-TiO_2_ on bivalve feeding and physiology is still scarce.

The ocean surface pH now is approximately 0.1 units lower than the preindustrial epoch^24^ and a reduction of nearly 0.8 units within the next three centuries due to ocean acidification (OA) is predicted^24^,^25^. Some studies show that CO_2_-induced pH reduction impacts bivalve physiology by changing extracellular acid–base balance^26^,^27^, metabolic activities^28^ and feeding^29^,^30^. A rise in pCO_2_ levels can induce changes in the extracellular acid–base balance that can produce metabolic disturbances, adversely affecting relevant biological processes, such as calcification, metabolism, growth and fitness^31^,^32^. Many marine calcifying organisms have exhibited negative responses to high pCO_2_, such as disorder in metabolic rates^26^,^28^, reduction of food uptake^29^,^30^ and alteration in calcification and development^33^. Bivalve molluscs have a limited capacity for acid–base regulation due to the lack of developed ion-exchange and nonbicarbonate mechanisms^32^. Consequently, their ability to regulate the acid–base status of internal tissues during contact with low pH water is limited. In order to correct acid–base imbalances an organism must employ energetically costly active ion exchange mechanisms^32^. Furthermore, the extracellular alterations caused by exposure to elevated pCO_2_ are likely to affect processes such as energy partitioning and metabolism^32^,^34^.

Scope for growth (SFG), calculated from clearance rate, respiration rate and excretion rate, is a reliable tool for evaluating effects of environmental stressors on marine bivalves^35^,^36^,^37^,^38^. These physiological parameters not only present key processes related to energy budget, but also mirror the physiological plasticity of the animals to adapt stressful surrounding conditions^37^,^38^,^39^,^40^. However, toxic effects of nanomaterials on physiological responses in marine mussels receive little attention.

The bioaccumulation, environmental and biological impacts of nanoparticles are difficult to predict because of the complexity of marine ecosystem and lack of relevant information to date^1^. Further complexity is expected when interactions of metal oxide nanoparticles with seawater pH are present as pH change in water is predicted to influence metal speciation^41^ and nanoparticle behavior (e.g., aggregation and disaggregation)^42^. It is reported that the size and form of nanoparticles can influence the toxicity on aquatic organisms^13^. For instance, the bulk form of nano-TiO_2_ is more toxic with respect to histopathological and histochemical changes^5^. Thus, nano-TiO_2_ may exert different toxic effect on mussels if their behaviour was changed by CO_2_-induced pH reduction. Despite meriting considerable research effort in recent years, the biological impacts of nanoparticle or reduced seawater pH have been mostly considered with single factor exposure in marine environmental research, whereas their interactive effects are still poorly understood^41^. For instance, to date, whether the toxicity of nano-TiO_2_ on marine animals is affected by pH is still unclear. As nanoparticles and CO_2_-induced pH reduction may occur simultaneously and the physiological responses of the animals subjected to such multiple stressors are unclear, their interactive effects must be thoroughly clarified in marine bivalves^41^. Recent evidences suggest that interactions of nano-TiO_2_ with other chemical/physical factors may cause an increased toxicity or adverse effects in different marine species, for example, 2,3,7,8-TCDD, Cd^2+^ and hypoxia increased the toxicity of nano-TiO_2_ to marine mussels, raising attentions on ecotoxicological effects of nano-TiO_2_ on aquatic organisms^3^,^4^,^21^,^43^,^44^. Consequently, it is essential to explore how the combination of NP and reduced seawater pH affects marine organisms.

The thick shell mussel *Mytilus coruscus* is an ecologically important marine bivalve, widely inhabiting in the Changjiang estuary and coastal waters of the East China Sea. Our sampling area, the Shengsi island, is the largest area for mussel aquaculture in China, where in recent years mass mussel mortalities have been found due to hypoxia or some other environmental stressors, for example, large pH fluctuation in summer. A large amount of nutrients from estuaries of Changjiang and Qiantangjiang has entered this area, resulting in eutrophication and biological CO_2_ production. Consequently, in these regions, organisms can experience metal pollutants and pH fluctuations due to runoff events, eutrophication and biological CO_2_ production. Hence, this species is a typical organism for reflecting the biological effects of NP and pH change. Titanium particles have been reported to be released into some aquatic environments with concentrations of 0.1~3 mg/L, representing an environmental exposure and a threat for aquatic organisms and ecosystem health^45^,^46^. In our earlier studies, nano-TiO_2_ ranged from 2.5–10 mg/L in seawater caused some immune toxic effects on hemocytes in mussels^44^. Moreover, high concentrations of nano-TiO_2_ (10 mg/L) resulted in moderate gill damage^5^. Hence, the physiological functions, such as filtration and respiration, probably are affected by such TiO_2_ concentration. Moreover, whether high pCO_2_ can affect mussel’s ability to cope with nano-TiO_2_ has not yet been examined. We hypothesize that nano-TiO_2_ may be more toxic under reduced pH conditions compared to under normal pH conditions as pH reduction causes additional stress to the physiology of mussels. The purpose of this study was to assess the interactive effects of nano-TiO_2_ and high pCO_2_ on *M. coruscus* by integrating key eco-physiological parameters, i.e., clearance rate, food absorption efficiency, organic rate in faeces, respiration rate, ammonia excretion rate, oxygen to nitrogen (O:N) ratio as well as scope for growth (SFG). Such data are essential for the toxicity assessment of nano-TiO_2_ in coastal and estuarine areas (e.g., the Shengsi island), where much nutrients flow in and eutrophication and CO_2_-induced pH reduction frequently occur^47^.

## Results

### NPs characterization

X-ray diffraction patterns revealed the presence of both anatase (70%) and rutile (30%) phases (Supplementary Figure 1A), which was consistent with the product information nano-TiO_2_ showed different sizes under scanning electron microscope (SEM) and transmission electron microscope (TEM), the morphology was typical of grinded particles, and some particles were agglomerated (Supplementary Figure 1B and C). A size histogram of particles based on TEM image showed a relatively non-uniform composition with a majority of 20–30 nm particles (Supplementary Figure 1D), basically in agreement with the declared size.

### Seawater chemistry

Salinity of the water consistently was around 25‰, and dissolved oxygen in the exposure tanks consistently was maintained at about 7.0 mg l^−1^ during the experiment. Total alkalinity ranged from 2136 to 2276 μmol kg^−1^, and CO_2_ levels were maintained at ca. 350 μatm and ca. 2600 μatm in the normal (pH 8.1) and low pH (pH 7.3) treatments, respectively. The nano-TiO_2_ concentrations in seawater determined by atomic absorption spectrophotometer in different pH conditions, were in average 1.66 ± 0.18 mg l^−1^ and 7.88 ± 0.55 mg l^−1^ (pH 8.1) and 1.52 ± 0.14 mg l^−1^ and 7.37 ± 0.75 mg l^−1^ (pH 7.3) for the nominal 2.5 (low) and 10 mg l^−1^ (high) exposure concentration, respectively, and no significant effects of pH on nano-TiO_2_ concentrations were found. The detailed seawater chemistry in each treatment was summarized in Supplementary Table 1.

### Physiological parameters

**Figure 1:**
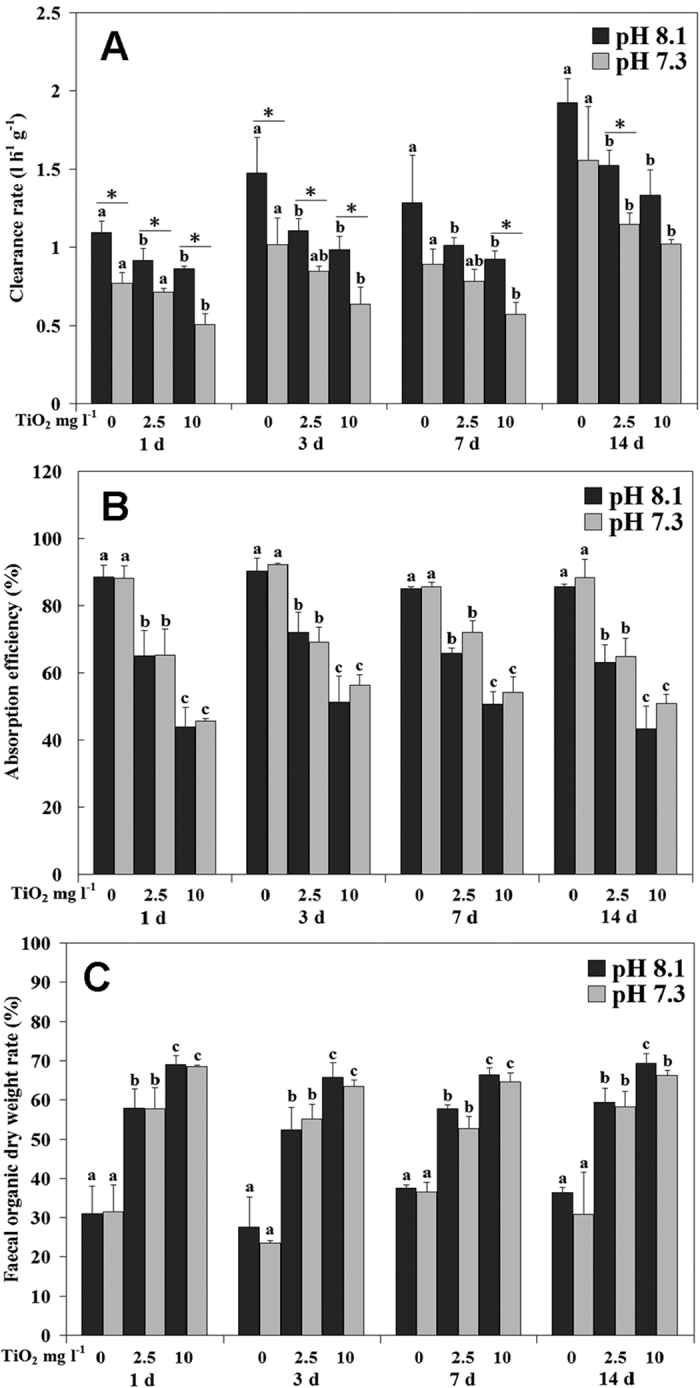
Clearance rate (CR, **A**), Absorption efficiency (AE, **B**) and faecal organic weight rate (E, **C**) of *M. coruscus* exposed to six treatments for 14 days. The values with different superscripts at each pH are significantly different among three nano-TiO_2_ treatments (P < 0.05). The values denoted by an asterisk between two pH groups at each n-TiO_2_ concentration are significantly different (P < 0.05).

Clearance rates were significantly decreased with the increased nano-TiO_2_ concentration with the lowest value observed at 10 mg l^−1^ nano-TiO_2_ treatment during the whole experiment at each pH level (Supplementary Table 2; Fig. 1A); pH significantly affected clearance rates at day 1 and 3 under all nano-TiO_2_ treatments, with lower values at pH 7.3 than pH 8.1; however, low pH only negatively affected clearance rates at day 7 under nano-TiO_2_10 mg l^−1^, and at day 14 under nano-TiO_2_ 2.5 mg l^−1^ (Supplementary Table 2; Fig. 1A).

Absorption efficiency significantly decreased with the increased nano-TiO_2_ concentration throughout the experiment at each pH level. However, pH showed no significant effect on AE during the whole experiment (Supplementary Table 2; Fig. 1B). Organic weight ratio in feces significantly increased with nano-TiO_2_ increment during the whole experiment at each pH level, but no pH effect was found on this parameter (Supplementary Table 2; Fig. 1C).

**Figure 2:**
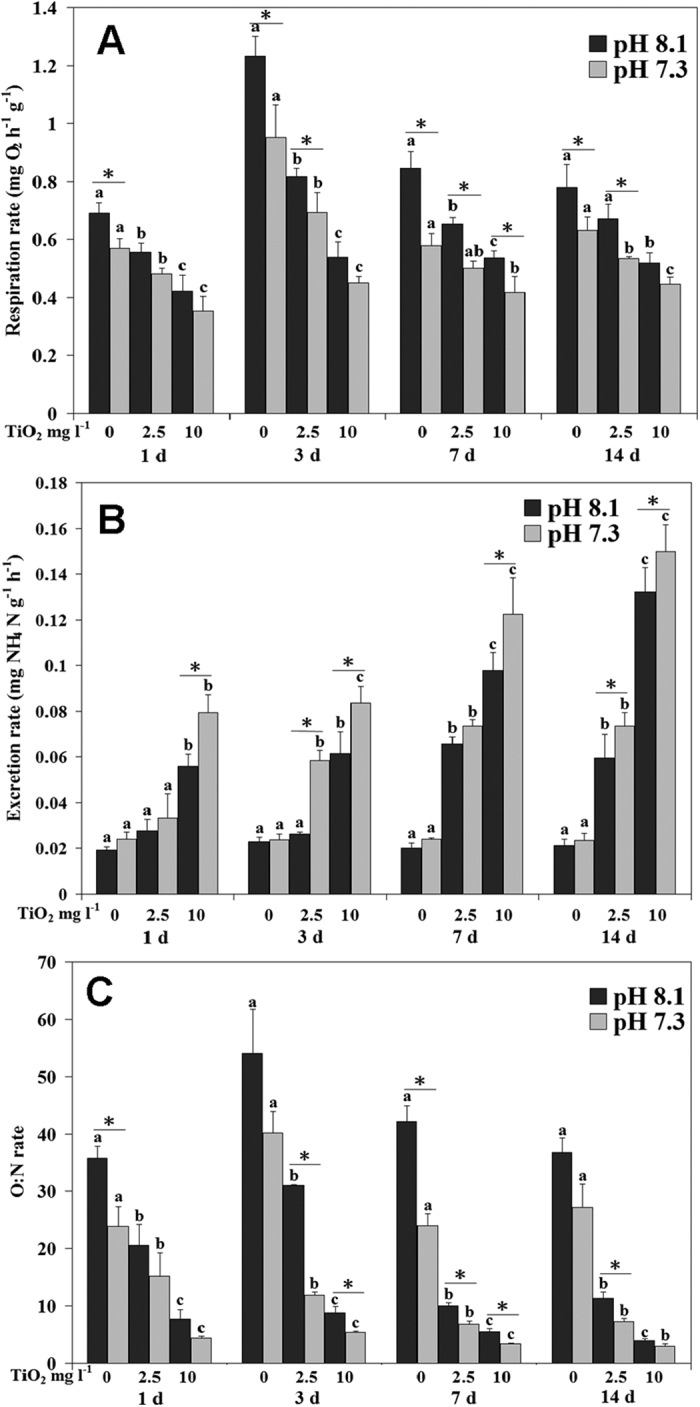
Ammonia excretion rate (ER, **A**), Respiration rate (RR, **B**) and O:N ratio (**C**) of *M. coruscus* exposed to six treatments for 14 days. The values with different superscripts at pH are significantly different among three nano-TiO_2_ treatments (P < 0.05). The values denoted by an asterisk between two pH groups at each nano-TiO_2_ concentration are significantly different (P < 0.05).

Respiration rates were significantly influenced by nano-TiO_2_ and pH during the entire experiment, and by their interactions at day 7 (Supplementary Table 2; Fig. 2A). Respiration rates decreased with nano-TiO_2_ increment during the experiment, with the lowest value observed under 10 mg l^−1^ nano-TiO_2_ condition. In general, low pH reduced the respiration rates, although sometimes there were no significant effects of low pH. At day 1, low pH only reduced respiration rates when nano-TiO_2_ was absent; at day 3 and day 14, low pH reduced respiration rates under nano-TiO_2_ 0 and 2.5 mg l^−1^ treatments; at day 7, low pH reduced respiration rates under all nano-TiO_2_ conditions (Supplementary Table 2; Fig. 2A).

Ammonia excretion rates were significantly influenced by nano-TiO_2_ and pH during the entire experiment, and by their interactions at day 1 and 3 (Supplementary Table 2; Fig. 2B). Excretion rates generally increased with nano-TiO_2_ increment, with the highest values observed at nano-TiO_2_10 mg l^−1^. For the pH effect, at day 1 and 7, excretion rate at pH 7.3 was significantly lower than pH 8.1 when mussels were exposed to nano-TiO_2_ 10 mg l^−1^; at day 3 and 14, pH 7.3 increased excretion rates of mussels under two nano-TiO_2_ treatments, respectively.

O:N ratios were significantly influenced by nano-TiO_2_ and pH during the experiment, and by their interactions at day 3, 7 and 14 (Supplementary Table 2; Fig. 2C). Generally, O:N ratio decreased with nano-TiO_2_ increment, with the lowest values observed at 10 mg l^−1^ nano-TiO_2_. For the pH effect, at day 1, O:N ratio of pH 7.3 was significantly lower than pH 8.1 when nano-TiO_2_ was absent. At day 3, O:N ratio of pH 7.3 was significantly lower than pH 8.1 under two nano-TiO_2_ conditions. At day 7, pH 7.3 reduced O:N ratio when nano-TiO_2_ was present. At day 14, O:N ratio of pH 7.3 was significantly lower than pH 8.1 when nano-TiO_2_ was 10 mg l^−1^ (Supplementary Table 2; Fig. 2C).

**Figure 3:**
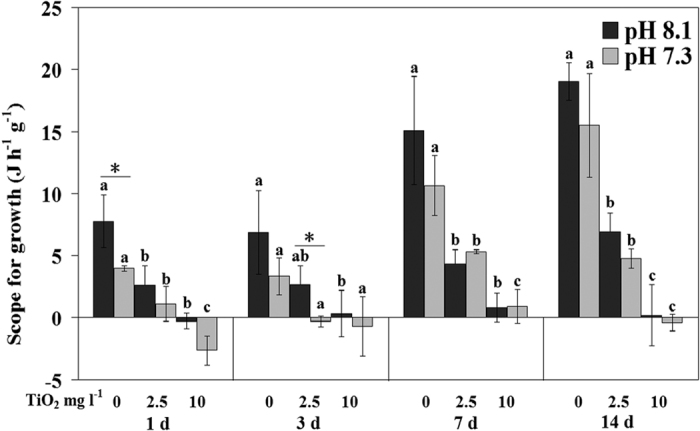
Scope for growth (SFG) of *M. coruscus* exposed to six treatments for 14 days. The values with different superscripts at each pH are significantly different among three nano-TiO_2_ treatments (P < 0.05). The values denoted by an asterisk between two pH groups at each nano-TiO_2_ concentration are significantly different (P < 0.05).

The scope for growth (SFG) values were affected by pH at day 1 and day 3, and by nano-TiO_2_ during the entire experiment, but there was no interactive effect of them (Supplementary Table 2; Fig. 3). SFG decreased with nano-TiO_2_ increment, and even negative values were observed under the highest nano-TiO_2_ concentration. However, pH only affected SFG at day 1 and 3. At day 1, when nano-TiO_2_ was absent, SFG of pH 7.3 was lower than pH 8.1. At day 3, SFG of pH 7.3 was significantly lower than pH 8.1 when nano-TiO_2_ was 2.5 mg l^−1^.

**Figure 4:**
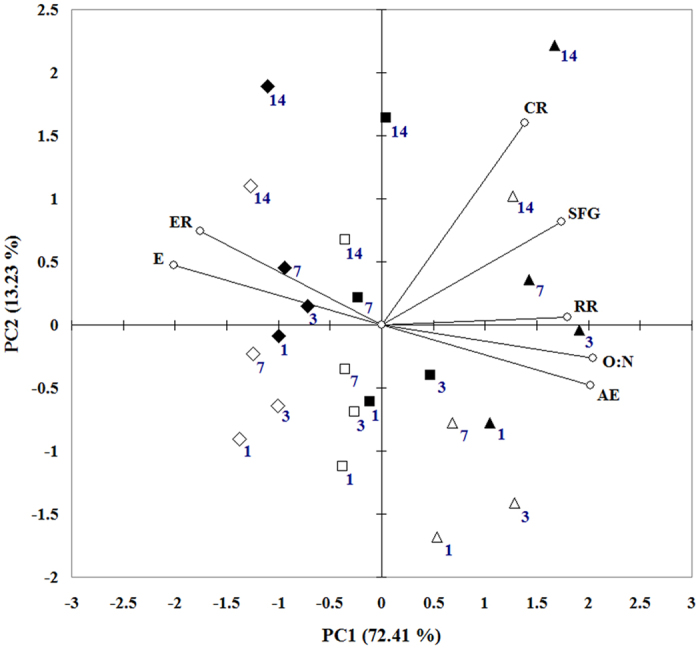
Principal component analysis (PCA) by integrating all measured parameters (CR, AE, RR, ER, O:N, SFG, E) for four times (days: 1, 3, 7 and 14) and six different treatments (▴–TiO_2_ 0 × pH 8.1, △–TiO_2_ 0 × pH 7.3, ▪–TiO_2_ 2.5 × pH 8.1, ▫–TiO_2_2.5 × pH 7.3, ♦–TiO_2_10 × pH 8.1, ◊–TiO_2_ 10 × pH 7.3) with a biplot. Both the scores of the experimental conditions and the loadings of the parameters (○) were present.

PCA showed that 85.64% of total variance was explained by PC1 (72.41%) and PC2 (13.23%) (Fig. 4). PC1 indicated a clear separation between non-nano-TiO_2_ and nano-TiO_2_ exposure treatments, showing a high convergence of most physiological parameters, especially high levels of SFG associated with CR and AE under non-nano-TiO_2_ treatments. PC2 separated two main experimental periods, i.e., day 1–7 and day 7–14. Generally, the reduced SFG with increased nano-TiO_2_ in this study was explained by inhibited physiological activities.

## Discussion

Doyle *et al*.^7^ found measurable concentrations of nano-TiO_2_ in the gills of mussel and oyster following exposure^7^. Adsorption of NPs on gill surfaces results in a number of sublethal effects, e.g., gill pathology (such as hyperplasia and edema), respiratory toxicity, oxidative stress and dietary stress^48^,^49^, which subsequently impair filtration and ingestion of mussels. In the present study, nano-TiO_2_ exposure reduced CR, hence the food ingestion rate. The food ingestion rate in the zebra mussels *Dreissena polymorpha* decreased greatly in nano-TiO_2_ exposure media^50^. In our experiment, the mussel valve opening, distinguishing whether *M. coruscus* was filtering was lower if nano-TiO_2_ was present, which was similar to *M. edulis* exposed to nano-polystyrene^51^. This indicates that *M. coruscus* was able to recognize nano-TiO_2_ and hence reduced its filtration. A decreased filtering activity might cause severe consequences if the mussels were exposed to such concentrations in natural environments. Continuous limitation of CR would impair food intake thereby growth if the mussels were subjected to long term exposure.

Some bivalve species have showed different CR responses to CO_2_-induced low pH. Fernández-Reiriz *et al*.^30^ reported a decreased CR in the clam *Ruditapes decussates* under high pCO_2_ conditions^30^. Liu and He (2012) found reduced CRs in both the mussel *Perna viridis* and the clam *Chlamys nobilis* under high pCO_2_ conditions^52^. In our study, CO_2_-induced low pH reduced the CR of *M. coruscus* during the first few days, afterwards CR was only negatively affected by low pH under nano-TiO_2_ conditions sometimes, probably because the mussels showed some adaptation to low pH along with time, but could not overcome the additional stressor, nano-TiO_2_. Similarly, the CR of *M. galloprovincialis* was unaffected by pH 7.5^37^. Sanders *et al*.^53^ also found that there was no significant effect of CO_2_-induced low pH on the filtering activity of king scallop *Pecten maximus*^53^. Thus, some bivalves can adapt reduced pH conditions, but additional stressors may change this situation based on the present study. It is known that some bivalves are sensitive to reduced pH under food limiting conditions^53^,^54^. Thus, in our study, nano-TiO_2_ first influenced the feeding and absorption of the mussels, which would further reduce the tolerance to high pCO_2_ because of the reduced energy uptake. Although the purpose of our study was not to address food limiting effects, nano-TiO_2_ posed a sub-optimal dietary state for the mussels during the exposure.

High pCO_2_ did not show significant effect on AE, implying the normal performance of the digestive systems of the mussels exposed to CO_2_-induced low pH conditions. This was in agreement with the gastropod *N. conoidalis* in which AE was also insensitive to low pH^55^. In such situations, the digestive enzymes may be relatively stable under the low pH conditions in this study. In the present study, reduced AE probably is caused by the decreased CR under nano-TiO_2_ exposure. NPs can attach to algal cells and form clusters, which can settle readily, resulting in a large decline of the algal concentration^56^,^57^,^58^. Hereby, the intake of algal cells was subsequently impaired. Moreover, exposure to nano-TiO_2_ induced oxidative stress and lysosomal membrane alteration in the digestive gland of mussels^9^,^10^. In addition, some other molecular and functional parameters of digestive gland can be affected by nano-TiO_2_ in mussels^12^,^59^. These results further verify the hypothesis that NPs can enter the digestive system which is a typical target for NP toxic effect in mussels. AE impairment, representing a significant stress response suggests that nano-TiO_2_ exposure may cause a serious harm to mussel health.

Some authors reported metabolic decline under high pCO_2_ and suggested that altered extracellular pH could cause these reductions^26^,^34^. Similar metabolic depressions have been observed in the clam *R. decussatus*^30^,^60^, the scallop *Chlamys nobilis*^52^, the mussel *M. chilensis*^61^, and the scavenging gastropod *Nassarius conoidalis*^55^ exposed to high pCO_2_ conditions. The above reports are consistent with the present study, where the respiration of mussel was significantly lowered at high pCO_2_ levels. Because carbon dioxide interacts with intra- and extracellular fluids, internally elevated CO_2_ levels may result in a respiratory acidosis^62^. In our study, lower respiration rates under nano-TiO_2_ exposure indicated that the metabolic activity was weakened by nano-TiO_2_. More particularly, the oxygen consumption of *M. coruscus* showed a strong sensitivity to nano-TiO_2_ during the entire experiment, indicating a limited capacity to adapt to NP exposure. Reduction in RR can be indirectly due to the disruption of ventilation by nano-TiO_2_. In mussels, CR and RR are linked as both occur by filtering water over the gills when their valves are open. Mussels are known to spend less time opening and filtering when there are contaminants present in the water as a behavioural response to avoid uptake of the contaminant. Hence the reduction in CR and RR could also be due to this behavioural response to nano-TiO_2_ rather than direct gill toxicity. Limited respiratory efficiency results in a growing mismatch between basic oxygen demand and oxygen supply and finally causes hypoxia and anaerobic metabolism^63^, which may be harmful to the mussels.

ER is considered as an index of high pCO_2_ stress in marine mussels^30^,^37^. An increase in ER may imply a drastic increment in the consumption of amino acids. Higher ER values under high pCO_2_ conditions were observed when nano-TiO_2_ was present, indicating nano-TiO_2_ affects the CO_2_ effect on ER. Under nano-TiO_2_ treatments, the inverse correlation between ER and O:N ratios indicates the enhanced protein catabolism and subsequently inhibited growth.

The O:N ratio is considered as an index of the nutritional state of the mussels by showing the metabolism of substrates. According to Widdows^36^ and Fernández-Reiriz *et al*.^30^, in mussels, O:N ratios of more than 30 usually indicate a catabolism of carbohydrates and lipids, whereas less than 30 suggest a protein catabolism mainly. Hereby, low O:N ratios are generally a sign of a stressed condition^64^. In this experiment, high pCO_2_ and nano-TiO_2_ treatments showed lower O:N ratios owing to the decreased respiration and enhanced protein metabolism as a consequence of energy demands.

In our study, the growth of *M. coruscus* was not negatively affected by high pCO_2_ at the end in terms of SFG. Similar to our study, SFG was stable or even increased under moderate pCO_2_ level in *M. galloprovincialis*^30^. The SFG values became negative when *M. coruscus* was exposed high nano-TiO_2_, probably as a result of a significant reduction in CR. Given that most of the other parameters measured would be affected by reduced filtration activity (valve opening time) of the mussels and hence time spent for feeding or respiration, nano-TiO_2_ obviously would impair the growth of mussels. The SFG results suggest that *M. coruscus* is not able to grow when nano-TiO_2_ is more than 10 mg l^−1^ in seawater, and nano-TiO_2_ (above 10 mg l^−1^) is thus stressful to the mussels.

PCA distinguished non-nano-TiO_2_ treatments from exposed treatments since non-nano-TiO_2_ treatments were grouped together at positive side whereas exposed treatments were grouped at negative side by PC1, reflecting higher values of AE, O:N, RR, CR and SFG were achieved under non-nano-TiO_2_ conditions whereas nano-TiO_2_ induced high ER and E. PC2 reflected the time change of most physiological parameters, as higher values of E, ER, CR and SFG were positive, corresponding to the experimental time, namely the later period, day 7 and day 14. By integrating ANOVA and PCA results, the characteristics of physiological responses to nano-TiO_2_ exposure were lower CR, AE, RR, O:N ratio and SFG associated with higher E and ER. CR, RR and AE can be reflected by filtration activity. In the present study, the filtration activity was impaired by nano-TiO_2_, hence the CR, RR and AE were reduced. SFG is mostly dependent on absorption rate, which is mostly determined by ingestion rate and AE. Thus, if CR was reduced, SFG was also reduced (Fig. 4). In contrast, higher E and ER under high nano-TiO_2_ indicate the low absorption rate and subsequently high protein catabolism, which can also impair the growth of mussels.

Nanoparticles exert toxicity via mainly oxidative stress mechanisms^65^. *M. edulis* increased oxyradical production and antioxidant enzyme activities when they were exposed to nano-TiO_2_^11^,^15^. In our previous study, the hemocyte functions of *M. coruscus* exposed to nano-TiO_2_ and high pCO_2_ were impaired associated with an increase of ROS production, indicating an oxidative stress and fitness impairment^66^. Hence, in the present study, some physiological functions, such as clearance, respiration and absorption were all impaired when mussels were exposed to nano-TiO_2_, especially the combined treatment. The CO_2_-induced low pH in seawater could change the physiochemical properties of nano-TiO_2_ and lead to a slightly greater aggregation of nano-TiO_2_^66^. In the present study, although the exposed nano-TiO_2_ concentration under low pH was lower than under normal pH, no significant difference was found in each nano-TiO_2_ concentration (Supplementary Table 1). It is reported that mussels are able to capture and ingest aggregates of nanoparticles more efficiently compared with those freely suspended^6^. Thereby, under CO_2_-induced low pH conditions, *M. coruscus* may accumulate more nano-TiO_2_ than pH 8.1, accordingly causing more severe toxic effects. Mussels usually lower their metabolic rate after exposure to acidified water^26^. In the present study, mussels closed their valves when nano-TiO_2_ was present, which further decreased their filtration and respiration. Thus, both high pCO_2_ and nano-TiO_2_ exerted negative effects on the physiological functions of mussels with more severe effects than the single stressor.

## Conclusions

High pCO_2_ showed minor effect on the physiology of *M. coruscus* as most physiological parameters were almost unaffected by CO_2_-induced low pH. In contrast, nano-TiO_2_ showed significant negative effects on the feeding and physiology of mussels, suggesting that nano-TiO_2_ may present a health threat to the mussels, as depressions in feeding and digestion may reduce the ability of mussels to grow and defense predators. Above all, most physiological functions of *M. coruscus* were more severely impaired by the combination of nano-TiO_2_ and high pCO_2_, suggesting a synergistic effect on mussels.

## Methods

### Experimental animals

Experimental mussels (30.0 ± 2.0 mm shell length, 70.0 ± 5.0 mg dry tissue weight) were collected from the Shengsi island of Zhejiang Province, China (water temperature: 25.0 °C; salinity: 25.0‰; and pH: 8.1). The experimental mussels were wild, and the handling of them was conducted in accordance with the guidelines set by the Institutional Animal Care and Use Committee (IACUC) of Shanghai Ocean University, Shanghai, China. After transportation to aquarium of the university, the mussels were held in fibre-glass tanks (500 l) with a filtering apparatus in laboratory. The laboratory conditions were maintained according to sampling area in September: temperature, salinity, dissolved oxygen and pH were kept constant at 25 ± 0.5 °C, 26 ± 1‰, 7 ± 0.5 mg O_2_ l^−1^ and 8.10 ± 0.02. Mussels were fed with the microalgae *Isochrysis galbana* daily (25,000 cells ml^−1^, ca. 3% of the tissue dry weight). Two weeks were allowed for mussels to acclimatize to the above laboratory conditions.

### Characterization of nano-TiO_2_

#### Preparation of nano-TiO_2_ solution

Nano-TiO_2_ P25 particles (anatase/rutile, 7/3, declared purity of >99.5%, ~21 nm, spec. surface area 35–65 m^2^/g) were purchased from Sigma Aldrich. Stock suspensions (10.0 g l^−1^) were prepared daily with sterile filtered seawater (0.45 μm Teflon filter, pH 8.1, salinity 25‰) by sonication at 100 W, 50% on/off cycle, for 15 min in a cooling ice bath-type sonicator (frequency 40 kHz, UP200S Hielscher Ultrasonic Technology, Teltow, Germany).

#### TiO_2_ characterization by X-ray diffraction and electron microscope

X-ray powder diffractometer (Siemens D500, Karlsruhe, Germany) was applied to exam the crystal structures of nano-TiO_2_. Scanning electron microscope (FEI/Philips XL30 Esem-FEG, Netherlands) and transmission electron microscope (FEI/Philips Tecnai 12 BioTWIN, the Netherlands) were used to observe the surface morphology, primary size and shape of nano-TiO_2_^44^. The diameter of 1000 NPs was measured using an image analysis program (Image J, v1.44; National Institute of Health, USA).

### Experimental design and system

The experimental mussels were randomly divided to six treatments with three nano-TiO_2_ concentrations (0 (control), 2.5 and 10.0 mg l^−1^) under two pH values (7.3, 8.1(control)). The pH 7.3 was selected as an extreme pH value expected by the year 2300 and relevant for some coastal waters^25^,^47^,^67^. The low pH treatment was achieved by aerating pure carbon dioxide. The experimental system included a computer, pH regulator (DAQ-M; Loligo^®^ Systems Inc., Tjele, Denmark), pH meter, solenoid valve, CO_2_ and air supplies, water pump, filtering system, temperature regulator, head tank and exposure tank. The CO_2_ flow from the pure CO_2_ cylinder to the header tank was controlled by the pCO_2_/pH regulator which can open or close the solenoid valve when the seawater pH values deviated from the preset values by ±0.1 pH units. Water temperature was kept at 25 °C by temperature regulators. The tanks were covered with acrylic sheets to prevent external interference. To achieve exposed concentrations of 2.5 and 10 mg l^−1^ nano-TiO_2_ in the experimental tanks, each test solution was prepared in a water tank (500 l) by adding relevant volume of 10.0 g l^−1^ sonicated stock solution to the seawater, respectively. The seawater in the exposure tank was renewed and re-dosed everyday with new working solutions of nano-TiO_2_ to keep the exposure at relatively consistent nano-TiO_2_ concentrations. In each treatment, there were three flow-through tanks (30 l) as three replicates (30 mussels per replicate), and mussels were fed with microalgae *Isochrysis galbana* (2.5 × 10^4^ cells ml^−1^) at 1^st^, 3^rd^, 5^th^, 7^th^, 9^th^,11^th^ and 14^th^ day during the whole experiment. The mussels were exposed to six treatments for 14 days, and examinations on all physiological parameters of *M. coruscus* were carried out on the 1^st^, 3^rd^, 7^th^ and 14^th^ day.

### Monitoring of carbonate chemistry of seawater

Gran titration was applied to measure total alkalinity (TA) with a total alkalinity titrator system^68^. The pH and TA were used to calculate other carbonate system parameters, i.e., partial pressure of CO_2_ (pCO_2_) and dissolved inorganic carbon (DIC), and the saturation state of omega calcite and aragonite using CO_2_SYS software as described previously^37^. Nanoparticle concentrations in water were measured using the standard test method for determination by atomic absorption spectroscopy of titanium dioxide content^69^.

### Physiological measurements

#### Clearance rate

Clearance rate (CR) was measured using all individuals as a whole in each replicate tank. Prior to CR measurement, the mussels were fasting for at least twelve hours to evacuate their digestive tracts. The initial microalgae concentration was 2.5 × 10^4^ cells ml^−1^, and no mussels produced pseudo-faeces at this concentration. Three identical tanks without experimental mussels were used as the control. An initial 20 ml water was sampled from the tank center with a syringe at the beginning, and then 20 ml aliquots were collected at 30 min intervals for 120 min, without being influenced by large decrease of algal concentration (<30%). The algal concentration in each sample was counted by a particle analyzer (Multisizer 3 Coulter Counter, Beckman, Irvine, USA). Cell concentrations in control tanks did not show any significant variation during the measurements. CR was calculated according to the formula of Coughlan^70^:


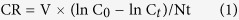


where CR represents the clearance rate (l h^−1^), V represents the water volume in the tank (l), C_0_ represents the initial microalgae concentration (cells ml^−1^), C_t_ represents the microalgae concentration at time t (cells ml^−1^), N represents the number of experimental mussels in the tank, t represents the sampling time (h). CR and the rest physiological parameters were standardized to unit dry weight (see below scope for growth).

Ingestion rate (IR) was computed by multiplying POM (particulate organic matter, mg l^−1^) by CR^71^, i.e., the food intake per hour. The POM concentration was transformed to joules using a conversion factor of 23.5 J mg^−1^ for *Isochrysis galbana*^72^.

#### Absorption efficiency

Absorption efficiency (AE) was determined according to Conover^73^. AE was determined by collecting the feces after CR measurements in each replicate tank. The organic content in microalgae was measured by filtering 5 L seawater with 2.5 × 10^4^ cell ml^−1^ algae using glass fiber filters (Whatman^®^ GF/C). Ammonium formate solution (0.5 M) was used to rinse these fulter papers which were then dried at 110 °C for 24 hours and weighed, and then ashed in a muffle furnace (450 °C for 6 hours) and reweighed. Blank GF/C filters were also treated using the same procedure to correct weight change caused by daily humidity variations. Faeces were carefully collected using a pipette from the tanks 12 hours after the CR measurements, and the organic content of faeces was determined as above. AE was calculated according to Conover^73^:


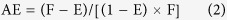


where AE represents the absorption efficiency (%), F represents the ratio of ash-free dry weight:dry weight in the microalgae, and E represents the ratio of ash-free dry weight:dry weight in the faeces.

#### Respiration rate

Fifteen mussels were randomly sampled from each tank and divided averagely into three replicates. The respiration rate (RR) was determined for five mussels in a closed glass respirometer (1000 ml) filled with air-saturated seawater from the corresponding experimental tank for one hour. To ensure the mussels started to respire in the chamber, the measurement began 20 minutes later when the mussels opened their valves. DO concentrations in three chambers without mussels were also recorded as the control. The oxygen consumption rate within the chamber was measured by oxygen meters (model YSI 58). The oxygen concentrations at the beginning and the end in each chamber were recorded.

The RR was calculated according to the following formula:


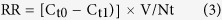


where RR represents the respiration rate (mg O_2_ h^−1^), C_t0_ and C_t1_ represent the initial and final DO concentrations in the chamber (mg O_2_ l^−1^), V (l) represent the volume of the seawater in the chamber, N represents the number of mussels in the chamber, t (h) represents the time elapsed. RR values were converted into J/h using a conversion coefficient of 13.98 J mg O_2_^−1 ^74.

#### Ammonia excretion rate

When RR measurements were finished, ammonia excretion rates (ER) of the same mussels were examined. Water was sampled from each chamber and stored at −20 °C until analysis. The concentration of ammonia produced by mussels was measured by the phenol-hypochlorite method^75^. ER was obtained from the difference of ammonia concentrations between the chamber containing mussels and the control chamber according to the equation:


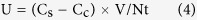


where U represents the rate of ammonia excretion (mg NH_4_–N h^−1^), C_s_ represents the ammonia concentration (mg l^−1^) in the experimental sample, C_c_ represents the ammonia concentration in the control sample, V represents the volume (l) of seawater in the chamber, N represents the number of mussels and t represents the time elapsed (h). Values of excretion rate were converted into J h^−1^ using a conversion coefficient of 25 J mg NH_4_–N^−1 ^76. The ratio of oxygen consumption to ammonia excretion expressed as atomic equivalents (O:N) was calculated to assess the utilization of different biochemical compositions for energy metabolism^36^.

#### Scope for growth

At the end of the experiment, soft tissues of the mussels were collected and dried at 90 °C for 24 h to calculate their tissue dry weight. CR (l h^−1^), RR (mg O_2_ h^−1^) and ER (mg NH_4_–N h^−1^) were converted to mass specific rates as a ‘standard mussel’ of 1 g dry weight using the formula: *Ys *= (*Ws*/*We*)^*b*^ × *Ye*, where *Ys* is the physiological value for a mussel of standard weight, *Ws* is the standard weight (1 g), *We* is the measured weight of the mussel (g), *Ye* is the uncorrected (measured) physiological value, and *b* is the weight exponent for the physiological value (b = 0.67). Each physiological parameter was transformed to energy equivalents (J h^−1^ g^−1^) to calculate the scope for growth (SFG), showing the difference between the energy acquired from the food and the energy depleted by respiration and excretion.

SFG was calculated according to Smaal and Widdows^74^,^76^,^77^:


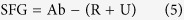


where SFG represents scope for growth (J h^−1^g^−1^), Ab represents absorption rate which derives from IR × AE (J h^−1^ g^−1^), R represents the energy loss in respiration (J h^−1^g^−1^), and U represents the energy loss in ammonia excretion (J h^−1^g^−1^).

### Statistical analyses

Normality of the data was checked by Shapiro-Wilk’s W test and homogeneity of variances was evaluated by Levene’s test using SPSS 16.0. To evaluate the interactive effects of pH and nano-TiO_2_ on physiological parameters, two-way repeated measures analysis of variance (ANOVA) were used. When there was a significant interaction, one-way ANOVA followed by Tukey’s HSD test or student t test was conducted for each factor separately in each level of the other factor. Principal component analysis (PCA) was conducted for multivariate analysis using XLSTAT^®^2014. A biplot was graphed with both the measured variables and the observations. All data were expressed as the mean ± standard deviations, and the results were statistically significant at p < 0.05.

## Additional Information

**How to cite this article**: Hu, M. *et al*. CO_2_-induced pH reduction increases physiological toxicity of nano-TiO_2_ in the mussel *Mytilus coruscus. Sci. Rep.* **7**, 40015; doi: 10.1038/srep40015 (2017).

**Publisher's note:** Springer Nature remains neutral with regard to jurisdictional claims in published maps and institutional affiliations.

## Supplementary Material

Supplementary Information
