# Supplementary material for: CO2-induced pH reduction increases physiological toxicity of nano-TiO2 in the mussel Mytilus coruscus
Source: Sci Rep. 2017 Jan 5;7:40015. doi: 10.1038/srep40015 (PMC5215630; doi:10.1038/srep40015)
Supplement: Supplementary Information [file srep40015-s1.doc]

**CO_2_-induced pH reduction increases physiological toxicity of nano-TiO_2_ in the mussel *Mytilus coruscus***

Menghong Hu, Daohui Lin, Yueyong Shang, Yi Hu, Weiqun Lu, Xizhi Huang, Ke Ning, Yimin Chen, Youji Wang

**Supplementary Materials**

**Supplementary Table 1.** Seawater chemistry monitoring during the experiment (mean ± SE, n = 4). pH was monitored by the pH/CO_2_ system continuously during the experiment. Salinity, temperature and total alkalinity (TA) were determined at each sampling time. Partial pressure of CO_2_ (pCO_2_), dissolved inorganic carbon (DIC), saturation degrees for calcite (Ωcal) and aragonite (Ωara) were calculated based on the above parameters. Nano-TiO_2_ concentrations were measured using the standard test method for determination of titanium dioxide content by atomic absorption spectroscopy.

| Treatments | S | T | pH_T_ | TA | DIC | p CO_2_ | Ωcal | Ωara | Nano-TiO_2_ |
| --- | --- | --- | --- | --- | --- | --- | --- | --- | --- |
| pH*TiO_2_(mg l^-1^) |  | (ºC) |  | (μmol Kg^-1^) | (μmol Kg^-1^) | (μatm) |  |  | (mg l^-1^) |
| 8.1*0 | 25.0±0.4 | 25.0±0.2 | 8.12±0.01 | 2276±29 | 2013±21 | 357±6 | 5.24±0.18 | 3.34±0.12 | 0 |
| 8.1*2.5 | 25.1±0.2 | 25.0±0.4 | 8.13±0.02 | 2273±33 | 2003±18 | 344±12 | 5.35±0.30 | 3.41±0.19 | 1.66±0.18 |
| 8.1*10 | 25.0±0.5 | 25.1±0.4 | 8.13±0.02 | 2266±25 | 1997±15 | 346±15 | 5.33±0.25 | 3.40±0.16 | 7.88±0.55 |
| 7.3*0 | 25.1±0.4 | 24.9±0.3 | 7.32±0.03 | 2164±27 | 2191±22 | 2600±152 | 0.96±0.08 | 0.61±0.05 | 0 |
| 7.3*2.5 | 24.9±0.3 | 25.4±0.2 | 7.34±0.03 | 2169±27 | 2189±20 | 2507±123 | 1.01±0.08 | 0.65±0.05 | 1.52±0.14 |
| 7.3*10 | 25.0±0.6 | 25.5±0.3 | 7.32±0.03 | 2136±40 | 2162±34 | 2607±152 | 0.95±0.07 | 0.61±0.03 | 7.37±0.75 |

**Supplementary Table 2.** Two-way ANOVA results on effects of nano-TiO_2_ and pH on clearance rate (CR), absorption efficiency (AE), faecal organic weight ratio (E), respiration rate (RR), excretion rate (ER), O:N ratio, and scope for growth(SFG). nano-TiO_2_: 0, 2.5 and 10 mg l^–1^; pH: 8.1 and 7.3.

| Source |  | CR |  |  | AE |  |  | E |  |  | RR |  |  |
| --- | --- | --- | --- | --- | --- | --- | --- | --- | --- | --- | --- | --- | --- |
|  |  | pH | TiO_2_ | pH*TiO_2_ | pH | TiO_2_ | pH*TiO_2_ | pH | TiO_2_ | pH*TiO_2_ | pH | TiO_2_ | pH*TiO_2_ |
|  | df | 1 | 2 | 2 | 1 | 2 | 2 | 1 | 2 | 2 | 1 | 2 | 2 |
| 1 d | MS | 0.389 | 0.09 | 0.01 | 0 | 0.591 | 0 | 1.02E-05 | 0.303 | 7.08E-05 | 0.035 | 0.089 | 0.001 |
|  | F | 109.894 | 25.541 | 2.891 | 0.019 | 97.834 | 0.038 | 0.003 | 92.783 | 0.022 | 22.793 | 57.091 | 0.764 |
|  | P | <0.001 | <0.001 | 0.094 | 0.893 | <0.001 | 0.963 | 0.956 | <0.001 | 0.979 | <0.001 | <0.001 | 0.487 |
| 3 d | MS | 0.569 | 0.291 | 0.015 | 0.002 | 0.523 | 0.005 | 0.001 | 0.313 | 0.002 | 0.121 | 0.536 | 0.016 |
|  | F | 32.319 | 16.531 | 0.866 | 0.378 | 106.511 | 0.958 | 0.313 | 118.687 | 0.915 | 27.38 | 121.44 | 3.588 |
|  | P | <0.001 | <0.001 | 0.445 | 0.55 | <0.001 | 0.411 | 0.586 | <0.001 | 0.427 | <0.001 | <0.001 | 0.06 |
| 7 d | MS | 0.37 | 0.288 | 0.062 | 0.01 | 0.332 | 0.002 | 0.004 | 0.171 | 0.001 | 0.144 | 0.084 | 0.009 |
|  | F | 18.744 | 14.592 | 3.16 | 6.542 | 224.884 | 1.468 | 6.985 | 278.265 | 1.694 | 88.803 | 51.716 | 5.704 |
|  | P | 0.001 | 0.001 | 0.079 | 0.055 | <0.001 | 0.269 | 0.051 | <0.001 | 0.225 | <0.001 | <0.001 | 0.018 |
| 14 d | MS | 0.561 | 0.507 | 0.002 | 0.015 | 0.499 | 0.001 | 0.007 | 0.256 | 0.001 | 0.064 | 0.074 | 0.003 |
|  | F | 18.232 | 16.492 | 0.059 | 2.752 | 92.438 | 0.269 | 2.12 | 81.984 | 0.218 | 29.559 | 34.34 | 1.193 |
|  | P | 0.001 | <0.001 | 0.943 | 0.123 | <0.001 | 0.769 | 0.171 | <0.001 | 0.808 | <0.001 | <0.001 | 0.337 |
| Source |  | ER |  |  | O:N |  |  | SFG |  |  |  |  |  |
|  |  | pH | TiO_2_ | pH*TiO_2_ | pH | TiO_2_ | pH*TiO_2_ | pH | TiO_2_ | pH*TiO_2_ |  |  |  |
|  | df | 1 | 2 | 2 | 1 | 2 | 2 | 1 | 2 | 2 |  |  |  |
| 1 d | MS | 0.001 | 0.004 | 0 | 208.122 | 846.851 | 30.466 | 29.636 | 80.873 | 2.041 |  |  |  |
|  | F | 14.696 | 90.044 | 4.29 | 25.868 | 105.259 | 3.787 | 16.445 | 44.876 | 1.133 |  |  |  |
|  | P | 0.002 | <0.001 | 0.039 | <0.001 | <0.001 | 0.053 | 0.002 | <0.001 | 0.354 |  |  |  |
| 3 d | MS | 0.002 | 0.004 | 0 | 664.58 | 2460.689 | 95.538 | 28.755 | 45.298 | 2.6 |  |  |  |
|  | F | 52.212 | 127.834 | 13.252 | 53.847 | 199.376 | 7.741 | 6.814 | 10.735 | 0.616 |  |  |  |
|  | P | <0.001 | <0.001 | 0.001 | <0.001 | <0.001 | 0.007 | 0.023 | 0.002 | 0.556 |  |  |  |
| 7 d | MS | 0.001 | 0.012 | 0 | 272.859 | 1443.961 | 121.391 | 5.535 | 223.976 | 12.777 |  |  |  |
|  | F | 11.668 | 207.409 | 3.14 | 122.816 | 649.94 | 54.639 | 1.126 | 45.569 | 2.6 |  |  |  |
|  | P | 0.005 | <0.001 | 0.08 | <0.001 | <0.001 | <0.001 | 0.309 | <0.001 | 0.115 |  |  |  |
| 14 d | MS | 0.001 | 0.022 | 9.83E-05 | 107.016 | 1359.013 | 28.769 | 20.212 | 466.925 | 3.327 |  |  |  |
|  | F | 8.357 | 313.916 | 1.433 | 26.195 | 332.653 | 7.042 | 4.174 | 96.435 | 0.687 |  |  |  |
|  | P | 0.014 | <0.001 | 0.277 | <0.001 | <0.001 | 0.009 | 0.064 | <0.001 | 0.522 |  |  |  |


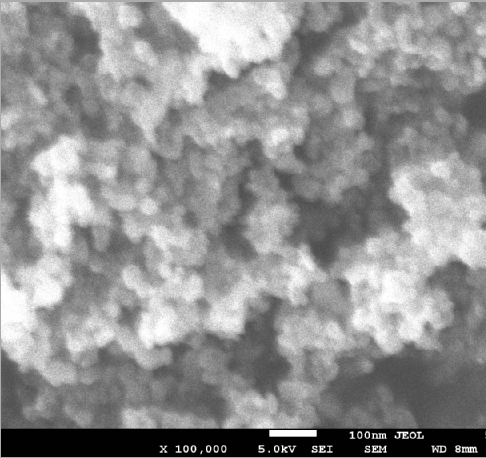


**A**

**B**


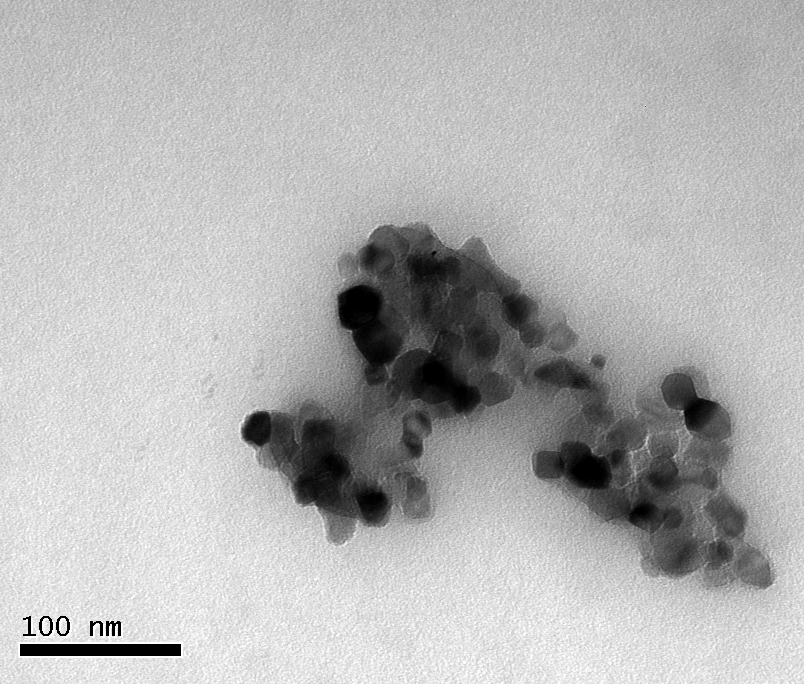

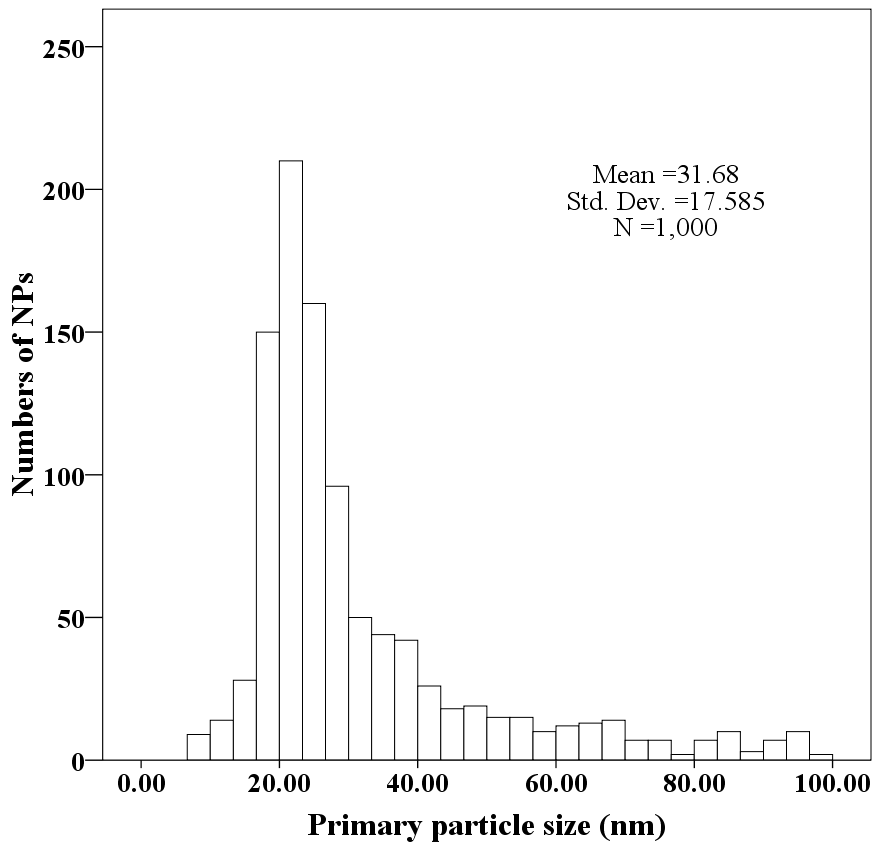


**D**

**C**

**Supplementary Figure 1.** Characterization of nano-TiO_2_. A, X-ray diffractograms pattern; B, SEM image; C, TEM image; D, Particle size distribution estimated by TEM images.
